# Quality, Reliability, and Content Characteristics of Metabolic Bariatric Surgery Videos on TikTok, YouTube, and Facebook: A Mixed-Methods Cross-Platform Analysis

**DOI:** 10.1007/s11695-026-08922-9

**Published:** 2026-09-11

**Authors:** Mohamed Yasser El-Mezayen, Ahmed Abokhozima, Nesma Saaed Ahmed, Passant Elaidy, Hossam Elghadban, Mohamed Shaker Abou Al-Ela, Noha Gamal AbdulNasser, Mohamed F. A. Kashwaa, Ahmed Abo Elmagd, Hassan El-Masry

**Affiliations:** 1 Faculty of Medicine, Alexandria University, Alexandria, Egypt; 2 MIAX Research Lab, Alexandria, Egypt; 3 Faculty of Medicine, Mansoura University, Mansoura, Egypt; 4 Surgery Department, Faculty of Medicine, Assiut University, Assiut, Egypt; 5 Faculty of Medicine, Ain Shams University, Cairo, Egypt

**Keywords:** Metabolic bariatric surgery, Social media, Video quality assessment, DISCERN, Global quality score, Video information and quality index

## Abstract

**Introduction:**

Social media platforms have become major sources of health information, including content related to metabolic bariatric surgery (MBS). While these platforms offer wide accessibility and high user engagement, concerns remain regarding the quality, reliability, and completeness of the information presented. Comparative evaluations across platforms integrating both quantitative quality assessment and qualitative insights are limited.

**Methods:**

A cross-sectional mixed-methods content analysis was conducted on 45 publicly available videos (15 per platform) from TikTok, YouTube, and Facebook, identified using standardized search terms. Data extraction included video metadata, engagement metrics, uploader characteristics, and content features. Video quality and reliability were assessed using the Video Information and Quality Index (VIQI), Global Quality Score (GQS), and DISCERN instrument. Inter-rater reliability was evaluated using intraclass correlation coefficients. Additionally, thematic analysis of rater comments was performed to explore qualitative content patterns.

**Results:**

Significant differences were observed across platforms in video duration, content characteristics, and engagement (*p* < 0.001). TikTok videos were shortest and demonstrated the highest median views and engagement rates, while YouTube videos were longest and Facebook videos showed moderate duration and engagement. Quality assessment revealed significantly lower VIQI, GQS, and DISCERN scores for YouTube compared with TikTok and Facebook (*p* < 0.001), with no significant difference between TikTok and Facebook. High-quality content (GQS ≥ 4) was absent on YouTube but present in 60–66.7% of TikTok and Facebook videos. Inter-rater reliability was good to excellent across all tools (ICC range: 0.81–0.88). Qualitative analysis demonstrated recurring themes of incomplete clinical information, limited discussion of risks, and a predominance of positively framed narratives, especially in short-form content.

**Conclusion:**

MBS-related content on social media varies significantly across platforms in terms of quality, reliability, and engagement. While TikTok and Facebook demonstrated higher overall quality scores than YouTube, all platforms exhibited important deficiencies in critical patient-centered information. High engagement does not equate to high informational quality. These findings highlight the need for improved content regulation, increased involvement of healthcare professionals, and development of standardized guidelines to enhance the educational value of bariatric surgery information on social media.

**Supplementary Information:**

The online version contains supplementary material available at https://doi.org/10.1007/s11695-026-08922-9.

## Introduction

Metabolic bariatric surgery (MBS) remains the most effective long-term treatment for severe obesity, producing substantial and sustained weight loss while improving obesity-related comorbidities, including type 2 diabetes mellitus, hypertension, and cardiovascular disease [1, 2]. As the number of bariatric procedures increases worldwide, patients are increasingly seeking information regarding surgical options, expected outcomes, risks, and postoperative lifestyle modifications [3]. Social media has emerged as one of the most influential sources of health information, enabling patients to access educational content, share personal experiences, and engage with healthcare communities [4, 5]. Platforms such as YouTube, Facebook, and TikTok are among the most frequently used social media platforms for health-related information seeking and have become important channels through which MBS information is disseminated [4, 5]. The accessibility and wide reach of these platforms provide opportunities for patient education but also raise concerns regarding the accuracy, completeness, and reliability of the information presented [3, 5].

YouTube has long been used as a source of medical and surgical information through longer-form educational videos produced by healthcare professionals, institutions, and patients. However, the absence of formal peer review and content regulation has generated concerns regarding the quality and reliability of information available to viewers [4]. More recently, TikTok has experienced rapid growth as a platform for short-form video content, offering high levels of user engagement and information dissemination. Nevertheless, the brief and highly visual nature of TikTok content may encourage oversimplification of complex medical topics, while much of the available information is produced by non-specialists. Facebook continues to serve as a major platform for health communication and patient support, but limited research has examined the quality and reliability of MBS information available on the platform [6]. These concerns have also been highlighted by the evolving approach of major social-media companies to content moderation. In January 2025, Meta CEO Mark Zuckerberg announced changes to the company’s content-moderation policies, emphasizing greater reliance on user-generated community notes and a reduction in certain forms of content moderation. Such developments underscore the ongoing tension between reducing censorship and ensuring the accuracy and reliability of information disseminated through social-media platforms [7].

The quality of online MBS information has important clinical implications. Previous research has demonstrated that educational videos can significantly improve patient understanding during the informed consent process and enhance shared decision-making [3]. Therefore, social media content may influence patient expectations, perceptions of surgical risks and benefits, and decisions regarding treatment. However, high levels of engagement do not necessarily indicate high-quality information. Studies evaluating bariatric surgery–related content on YouTube and TikTok have reported substantial variability in quality and reliability, and have shown that engagement metrics such as views, likes, and shares are often poorly correlated with validated measures of informational quality [3, 6].

Although previous studies have assessed MBS content on individual social media platforms, comparative evaluations across multiple platforms remain limited. Furthermore, most investigations have relied exclusively on quantitative quality assessment tools, providing limited insight into recurring content patterns and contextual factors that may influence educational value. Therefore, this study aimed to evaluate and compare the quality, reliability, content characteristics, and audience engagement of MBS videos on TikTok, YouTube, and Facebook using validated assessment instruments and complementary qualitative thematic analysis. By identifying platform-specific strengths and limitations, this study seeks to provide evidence that may inform patient education strategies and improve the dissemination of accurate MBS information through social media.

## Methods

### Study Design

This cross-sectional observational content analysis was conducted to evaluate the quality, reliability, and audience engagement of publicly available social media videos related to bariatric surgery. The study examined content available on three major social media platforms and assessed both informational quality and user engagement characteristics. Because the study involved analysis of publicly accessible online content and did not include human participants, patient data, or identifiable personal information, institutional review board approval was not required according to local regulations governing research involving publicly available data.

### Platform Selection

TikTok, YouTube, and Facebook were selected for analysis because they represent major sources of online health information and differ substantially in content format, audience characteristics, and communication style. TikTok primarily hosts short-form video content, whereas YouTube and Facebook support longer educational and informational videos. Together, these platforms provide a broad representation of contemporary social media environments through which patients may obtain information regarding bariatric surgery.

### Search Strategy and Video Selection

Video retrieval was performed on 13 January 2026 using a dedicated browser profile without prior browsing history, search history, or account personalization. Searches were conducted independently on TikTok, YouTube, and Facebook using the terms “bariatric surgery,” “gastric sleeve,” “gastric bypass,” and “weight loss surgery.” Search results were reviewed according to each platform’s default relevance ranking to reflect the information environment encountered by typical users.

Duplicate videos identified across search terms within the same platform were removed. Videos were eligible for inclusion if they primarily focused on bariatric surgery, were available in English or contained English subtitles, were publicly accessible at the time of retrieval, and had accumulated at least 1,000 views. Videos were excluded if they represented duplicate uploads from the same creator, consisted solely of promotional or advertising content without educational information, were inaccessible at the time of data collection, or were live-stream recordings that were not available as complete videos. For each platform, the first 15 eligible videos meeting the inclusion criteria were included, resulting in a final sample of 45 videos comprising 15 TikTok videos, 15 YouTube videos, and 15 Facebook videos.

### Data Extraction

A standardized data extraction form was developed prior to data collection. For each video, information regarding platform, title, upload date, duration, and URL was recorded. Engagement metrics, including total views, likes, comments, and shares when available, were collected at the time of retrieval. Uploader characteristics, including account name, follower or subscriber count, verification status, and uploader type, were documented. Uploaders were categorized as healthcare professionals, healthcare institutions or hospitals, official media organizations, self-media creators, or other content producers. For subsequent analyses, uploaders were additionally classified as professional or non-professional.

Content characteristics were also recorded, including video category, primary educational purpose, and intended audience. Videos were categorized as educational, patient experience, procedure demonstration, news or media content, or other. The presence of a healthcare professional on screen, visual aids, on-screen text, and spoken explanations was documented. Overall video tone was classified as positive, neutral, or negative. A complete list of extracted variables is provided in Table 1.

**Table 1:** Data Extraction Variables

Domain	Variables Extracted
Video Metadata	
Platform	TikTok, YouTube, or Facebook
Title	Full title as displayed
Upload date	Day/month/year
Duration	Total length in seconds
URL	Full video URL for verification
Engagement Metrics	
Total views	Cumulative view count at time of retrieval
Views per 30 days	Estimated 30-day rolling view rate
Total likes / likes per 30 days	Cumulative and rolling likes
Total comments / comments per 30 days	Cumulative and rolling comments
Total shares / shares per 30 days	Cumulative and rolling shares (where available)
Uploader Characteristics	
Uploader ID / channel name	As displayed on platform
Number of followers / subscribers	At time of retrieval
Certification/verification status	Verified or Not verified (platform badge)
Uploader type	Self-media, Hospital, Healthcare Institution, Official media, Doctor/Individual Medical Professional, Media Organisation, Media Personality
Professional status	Professional (healthcare organisation or physician) vs. Non-professional (lay/self-media creator)
Content Characteristics	
Video category	Patient experience, Educational, Procedure/Transformation Demonstration, News/media, Other
Primary educational aim	As coded from content (e.g., Outcomes and expectations, Procedure explanation, General awareness, Nutrition and diet)
Secondary educational aim	Where applicable
Target audience	General public, Mixed, Healthcare students/professionals, Specific patient subgroup
Video Features	
Medical professional on screen	Yes / No
Visual aids used	Yes / No (e.g., animations, diagrams, slides)
On-screen text used	Yes / No (captions, subtitles, overlaid text)
Spoken explanation	Yes / No (narration or verbal description of content)
Overall tone	Positive, Neutral, or Negative

All variables were coded independently by each rater. Discrepancies were resolved by consensus discussion

### Quality Assessment

Video quality was evaluated using the Video Information and Quality Index (VIQI) and the Global Quality Score (GQS). The VIQI assesses four domains: flow of information, accuracy of information, overall quality, and precision. Each domain is scored using a five-point Likert scale, with higher scores indicating superior informational and production quality. A composite VIQI score was calculated as the mean of the four domain scores [8].

The GQS was used as a global measure of educational quality and usefulness. This instrument consists of a single five-point item that provides an overall assessment of the quality of health-related video content. Higher scores indicate greater educational value and usefulness to viewers [9].

The DISCERN instrument was used to assess the reliability and completeness of treatment-related health information. DISCERN consists of 15 items evaluating the quality of information presented regarding treatment choices, together with a single overall quality rating item. Each item is scored on a five-point scale, with higher scores reflecting more reliable and comprehensive information. Mean DISCERN scores and mean overall DISCERN ratings were calculated for each video [10].

### Rater Training and Assessment

All videos were independently evaluated by four raters, each with more than 10 years of experience in bariatric surgery. Prior to formal assessment, raters participated in a structured calibration exercise using pilot videos that were not included in the final sample and provided with a rating guide contains all data regarding the rating criteria and tools attached to supplementary file. During this process, scoring criteria and operational definitions for the VIQI, GQS, and DISCERN instruments were reviewed to ensure consistency in interpretation and application. Following calibration, raters independently evaluated all included videos and remained blinded to the assessments of other raters throughout the study.

### Inter-Rater Reliability

Inter-rater reliability was assessed using the intraclass correlation coefficient (ICC), calculated using a two-way random-effects model with absolute agreement. This model was selected because all videos were independently assessed by multiple raters and absolute agreement between raters was of primary interest. ICC values were interpreted according to established recommendations, with values below 0.50 indicating poor reliability, values between 0.50 and 0.75 indicating moderate reliability, values between 0.75 and 0.90 indicating good reliability, and values greater than 0.90 indicating excellent reliability. Fleiss’ kappa statistics were additionally calculated for categorical variables.

### Derived Variables

Composite quality measures were calculated by averaging ratings across raters. The VIQI composite score was calculated as the mean of the four VIQI domains and subsequently averaged across all raters. The GQS score was calculated as the mean overall GQS rating across raters. Mean DISCERN scores were calculated by averaging the 15 DISCERN items and subsequently averaging across raters.

Total engagement was calculated as the sum of likes, comments, and shares. Engagement rate was calculated by dividing total engagement by total views, thereby normalizing engagement according to audience reach. Video age was defined as the number of days between the upload date and the date of data collection.

### Qualitative Content Analysis

In addition to quantitative assessment, a qualitative analysis of free-text rater comments was conducted to explore contextual aspects of video content that were not fully captured by the VIQI, GQS, and DISCERN instruments. The qualitative corpus comprised comments recorded by raters during video assessment, including observations regarding content characteristics, educational value, information completeness, presentation style, and perceived strengths or limitations of individual videos.

Thematic analysis was performed using an inductive approach based on the framework described by Braun and Clarke [11]. All substantive comments were reviewed repeatedly to achieve familiarity with the data, after which initial codes were generated and grouped into broader themes. Themes were iteratively reviewed and refined to ensure coherence and relevance to the study objectives. Particular attention was given to recurring patterns across videos and platforms, including information completeness, educational content, promotional characteristics, patient-centered communication, and disclosure of risks and treatment alternatives.

To enhance the credibility of findings, comments from multiple raters were examined for convergence and divergence of observations. Qualitative findings were subsequently integrated with quantitative results to provide additional context and facilitate interpretation of platform-specific differences in video quality and informational content.

### Measures to Reduce Bias

Several measures were implemented to reduce potential sources of bias. All searches were conducted using a clean browser profile without prior browsing history or account personalization to minimize algorithmic influence on search results. The same search terms, retrieval procedures, and eligibility criteria were applied consistently across all platforms. Raters underwent calibration prior to formal assessment and remained blinded to one another’s evaluations throughout the study. All videos were identified and assessed within a single data collection period to reduce temporal variation in content availability and engagement metrics.

### Statistical Analysis

Statistical analyses were performed using R software (version 4.4.1; R Foundation for Statistical Computing, Vienna, Austria) and IBM SPSS Statistics (version 26.0; IBM Corp., Armonk, NY, USA) [12, 13]. Continuous variables were summarized using means and standard deviations or medians and interquartile ranges, as appropriate, while categorical variables were reported as frequencies and percentages. Statistical significance was defined as a two-sided p-value < 0.05.

Because quality scores and engagement metrics demonstrated non-normal distributions, non-parametric statistical methods were used for inferential analyses. Comparisons among TikTok, YouTube, and Facebook were performed using the Kruskal–Wallis test. When statistically significant differences were identified, pairwise Mann–Whitney U tests with Bonferroni adjustment were conducted for post hoc comparisons. Effect size for pairwise comparisons was reported using rank-biserial correlation.

Categorical variables were compared using the chi-square test or Fisher’s exact test when expected cell counts were less than five. Effect size for categorical analyses was reported using Cramér’s V. Associations between quality scores, engagement metrics, and video characteristics were assessed using Spearman’s rank correlation coefficient.

## Results

### Sample Characteristics and Video Metadata

A total of 45 videos were analyzed: 15 from TikTok, 15 from YouTube, and 15 from Facebook Supplementary Table S1. Video duration differed significantly across platforms (*H* = 26.18, *p* < 0.001). TikTok videos were the shortest (mean 13.9 ± 6.0 s; median 12.0 s, IQR 9.5–18.0 s), followed by Facebook (mean 168.9 ± 239.7 s; median 108.0 s) and YouTube (mean 899.0 ± 1485.9 s; median 301.0 s). TikTok vs. YouTube: *U* = 0, *p*Bonf < 0.001, *r* = 1.00; TikTok vs. Facebook: *p*Bonf = 0.002, *r* = 0.73; YouTube vs. Facebook: *p*Bonf = 0.051.

All TikTok uploaders were unverified self-media creators (100%; 0% professional). All YouTube and Facebook uploaders were professional verified healthcare organizations or physicians (100%). All TikTok videos depicted patient-experience content with a positive tone, targeting the general public. YouTube content was predominantly educational (87%). A single negative-tone video, a news documentary on adverse bariatric surgical outcomes, appeared in the Facebook sample Table 2.

**Table 2:** Baseline Characteristics of Videos by Platform (*N* = 45)

Characteristic	TikTok (*n* = 15)	YouTube (*n* = 15)	Facebook (*n* = 15)	Overall (*N* = 45)
Video Metrics				
Duration, mean ± SD (s)	13.9 ± 6.0	899.0 ± 1485.9	168.9 ± 239.7	360.6 ± 876.4
Duration, median [IQR] (s)	12.0 [9.5–18.0]	301.0 [140.5–755.0]	108.0 [45.5–186.0]	—
Total views, median [IQR]	3,000,000 [1,650,000–5,100,000]	859,287 [496,794–1,185,408]	48,000 [16,000–62,000]	802,000
Total engagement, median [IQR]	305,300 [140,250–442,450]	8,965 [1,600–11,800]	147 [52–571]	14,700
Engagement rate, median [IQR]	10.3% [5.9–13.0%]	0.64% [0.41–1.05%]	0.30% [0.23–0.93%]	—
Followers, median [IQR]	32,300 [19,100–69,000]	56,500 [30,500–3,666,500]	22,500 [7,400–37,250]*	—
Uploader Characteristics				
Verified, n (%)	0 (0%)	15 (100%)	13 (87%)	28 (62%)
Professional, n (%)	0 (0%)	15 (100%)	15 (100%)	30 (67%)
Uploader type	Self-media (100%)	Hospital (53%) Official media (33%) Doctor (13%)	Healthcare institution (73%) Medical professional (13%) Other (13%)	—
Content Characteristics				
Video category	Patient experience (100%)	Educational (87%) Other (13%)	Educational (47%) Patient exp. (20%) Other (33%)	—
Tone: Pos / Neutral / Neg	15 / 0 / 0	9 / 5 / 0	9 / 3 / 1	33 / 8 / 1

Engagement = total likes + comments + shares. Engagement rate = total engagement / total views. Professional = hospital, healthcare institution, official medical media, or individual physician. *, Available only for 11 videos

### Inter-Rater Reliability

Reliability was evaluated using the Intraclass Correlation Coefficient (ICC, model 2,1) to assess absolute agreement among the four independent raters. ICC values demonstrated excellent reliability for the VIQI composite (ICC = 0.88) and GQS (ICC = 0.84), and good reliability for DISCERN items (ICC = 0.81).

### Video Content Features

Medical professional on screen differed significantly across platforms (*χ²* = 24.12, df = 2, *p* < 0.001): absent in all TikTok videos (0%, 0/15), present in 46.7% of YouTube (7/15), and 92.3% of Facebook videos (12/13). Spoken clinical explanation showed an identical gradient: 0% TikTok, 86.7% YouTube, 100% Facebook (*χ²* = 35.75, *p* < 0.001). Visual aids were used in all TikTok videos (100%), 60.0% of YouTube, and 15.4% of Facebook (*χ²* = 23.61, *p* < 0.001). On-screen text: TikTok 86.7% vs. YouTube 40.0% vs. Facebook 46.2% (*χ²* = 11.50, *p* = 0.022; Table 3).

**Table 3:** Video Content Features by Platform

Feature	TikTok *n*/*N* (%)	YouTube *n*/*N* (%)	Facebook *n*/*N* (%)	χ² (df)	*p*
Medical professional on screen	0/15 (0%)	7/15 (46.7%)	12/15 (80%)	24.12 (2)	< 0.001
Visual aids used	15/15 (100%)	9/15 (60.0%)	2/15 (13.3%)	23.61 (4)	< 0.001
On-screen text used	13/15 (86.7%)	6/15 (40.0%)	6/15 (40%)	11.50 (4)	0.022
Spoken explanation	0/15 (0%)	13/15 (86.7%)	13/15 (86.7%)	35.75 (2)	< 0.001

### Video Information Quality

#### VIQI Score

**Fig. 1:**
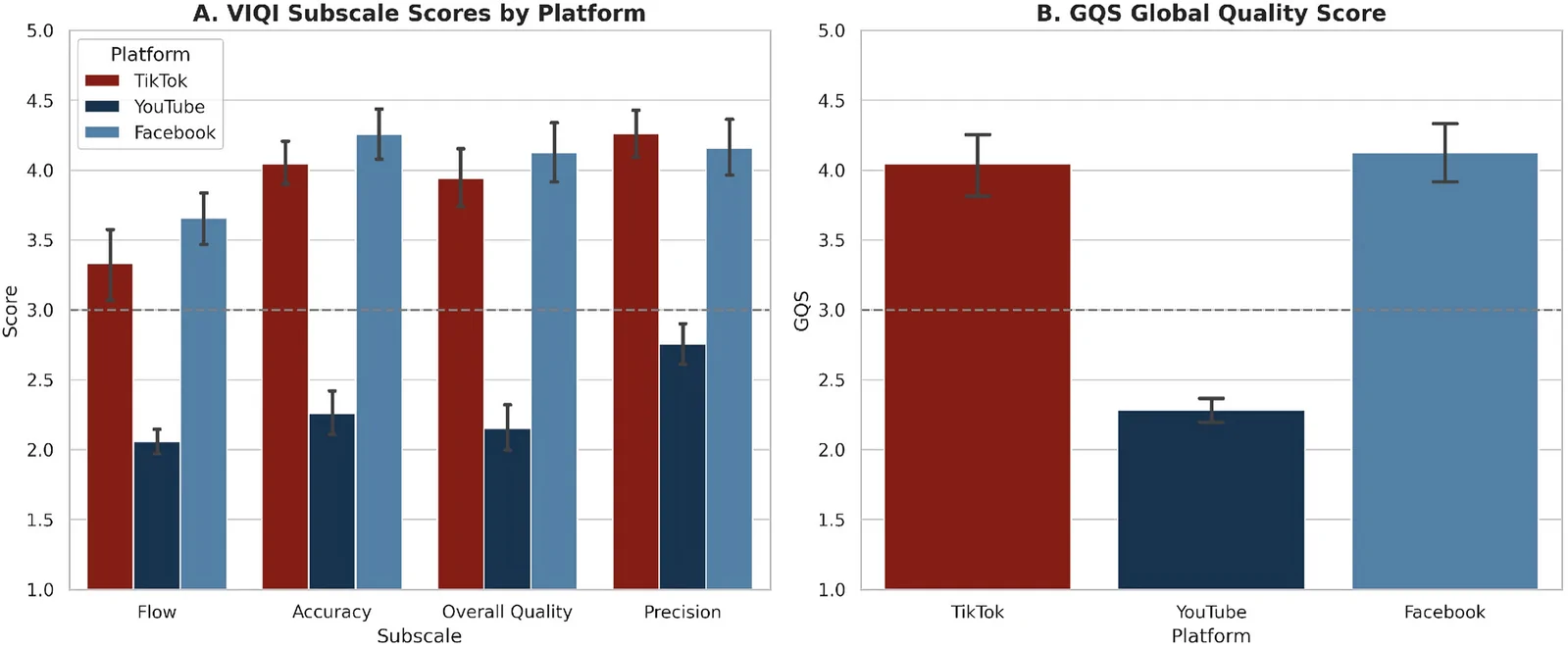
Panel **A**: Mean VIQI subscale scores by platform with SEM error bars. Panel **B**: Mean GQS scores by platform with significance brackets (*** *p* < 0.001, Bonferroni-corrected). Dashed line indicates scale midpoint

The VIQI composite, comprising Flow, Accuracy, Overall Quality, and Precision, differed significantly across platforms (*H* = 29.29, *p* < 0.001). TikTok (mean 4.14 ± 0.47; median 4.33) and Facebook (mean 4.13 ± 0.51; median 4.22) were statistically equivalent (*U* = 106, *p*Bonf = 1.00, *r* = − 0.005) and both significantly outperformed YouTube (mean 2.33 ± 0.18; median 2.50) with maximal effect sizes (TikTok vs. YouTube: *U* = 225, *p*Bonf < 0.001, *r* = − 1.00; Facebook vs. YouTube: *U* = 0, *p*Bonf < 0.001, *r* = 1.00) (Fig. 1a) (Table 4). YouTube scored lowest on all subscales.

#### GQS Score (Global Quality Item)

The GQS global item (“How would you rate the overall quality and usefulness of this video?“) showed an identical pattern (*H* = 29.99, *p* < 0.001): TikTok mean 4.03 ± 0.49, Facebook 4.09 ± 0.54, YouTube 2.25 ± 0.13 (Fig .1b). TikTok and Facebook did not differ (*U* = 92, *p*Bonf = 1.00, *r* = 0.13); both exceeded YouTube (all *p*Bonf < 0.001). VIQI and GQS were very strongly correlated (*r*s = 0.934, *p* < 0.001) (Table 4), confirming convergent validity.

**Table 4:** Comparison of Video Quality Scores Across Platforms (VIQI, GQS, and DISCERN)

Scale / Item	TikTok (*n* = 15)	YouTube (*n* = 15)	Facebook (*n* = 15)	H (KW)	*p*
VIQI Score (4-Item, Range 1–5)					
Composite mean	4.14 ± 0.47 [4.33; 3.83–4.46]	2.33 ± 0.18 [2.50; 2.29–2.50]	4.13 ± 0.51 [4.22; 4.08–4.48]	29.29	< 0.001
Flow	3.33 ± 1.03	2.15 ± 0.30	3.75 ± 1.09	—	—
Accuracy	4.20 ± 0.75	2.18 ± 0.81	4.18 ± 0.78	—	—
Overall quality	4.05 ± 0.76	2.20 ± 0.93	4.09 ± 0.76	—	—
Precision	4.25 ± 0.83	2.77 ± 0.78	4.16 ± 0.82	—	—
High quality (≥ 4), n (%)	10 (66.7%)	0 (0%)	12 (80.0%)	—	—
GQS Score (1 Global Item, Range 1–5)					
GQS mean	4.03 ± 0.49 [4.00; 3.75–4.38]	2.25 ± 0.13 [2.25; 2.25–2.25]	4.09 ± 0.54 [4.25; 3.81–4.50]	29.99	< 0.001
High quality (≥ 4), n (%)	9 (60.0%)	0 (0%)	10 (66.7%)	—	—
DISCERN (Range 1–5)					
Overall rating	3.73 ± 0.56 [3.75; 3.25–4.13]	2.12 ± 0.35 [2.25; 2.13–2.25]	3.66 ± 0.53 [3.75; 3.50–4.00]	29.44	< 0.001
15-item subscale mean	3.16 ± 0.57	1.92 ± 0.10	3.26 ± 0.45	29.34	< 0.001
High quality (overall ≥ 4), n (%)	5 (33.3%)	0 (0%)	5 (33.3%)	—	—

*KW* Kruskal-Wallis. Bonferroni-corrected post-hoc Mann-Whitney U: TikTok and Facebook both exceeded YouTube on all composite scales (*r* ≥ 0.995, pBonf < 0.001); TikTok vs. Facebook non-significant (pBonf ≥ 0.56)

#### DISCERN Scores

The DISCERN overall rating differed significantly (*H* = 29.44, *p* < 0.001): TikTok 3.73 ± 0.56, Facebook 3.66 ± 0.53, YouTube 2.12 ± 0.35. TikTok and Facebook were equivalent (*U* = 104, *p*Bonf = 1.00); both exceeded YouTube (*p*Bonf < 0.001). DISCERN overall ≥ 4 (high quality): TikTok 5/15 (33.3%), Facebook 5/15 (33.3%), YouTube 0/15 (0%). The 15-item subscale mean showed the same pattern (TikTok 3.16 ± 0.57; Facebook 3.26 ± 0.45; YouTube 1.92 ± 0.10; *H* = 29.34, *p* < 0.001) (Table 4).

Despite the uniformly low proportion of videos meeting the predefined high-quality thresholds, the YouTube videos were not uniformly devoid of educational features. Most were classified as educational (13/15, 86.7%), with spoken clinical explanations present in 13/15 (86.7%), visual aids in 9/15 (60.0%), and a medical professional visible in 7/15 (46.7%). However, qualitative rater comments indicated that the videos generally emphasized postoperative weight loss and changes in body profile, with limited clinical context and incomplete discussion of complications, long-term outcomes, treatment risks, and alternative treatment options.

### DISCERN Item-Level Completeness

**Fig. 2:**
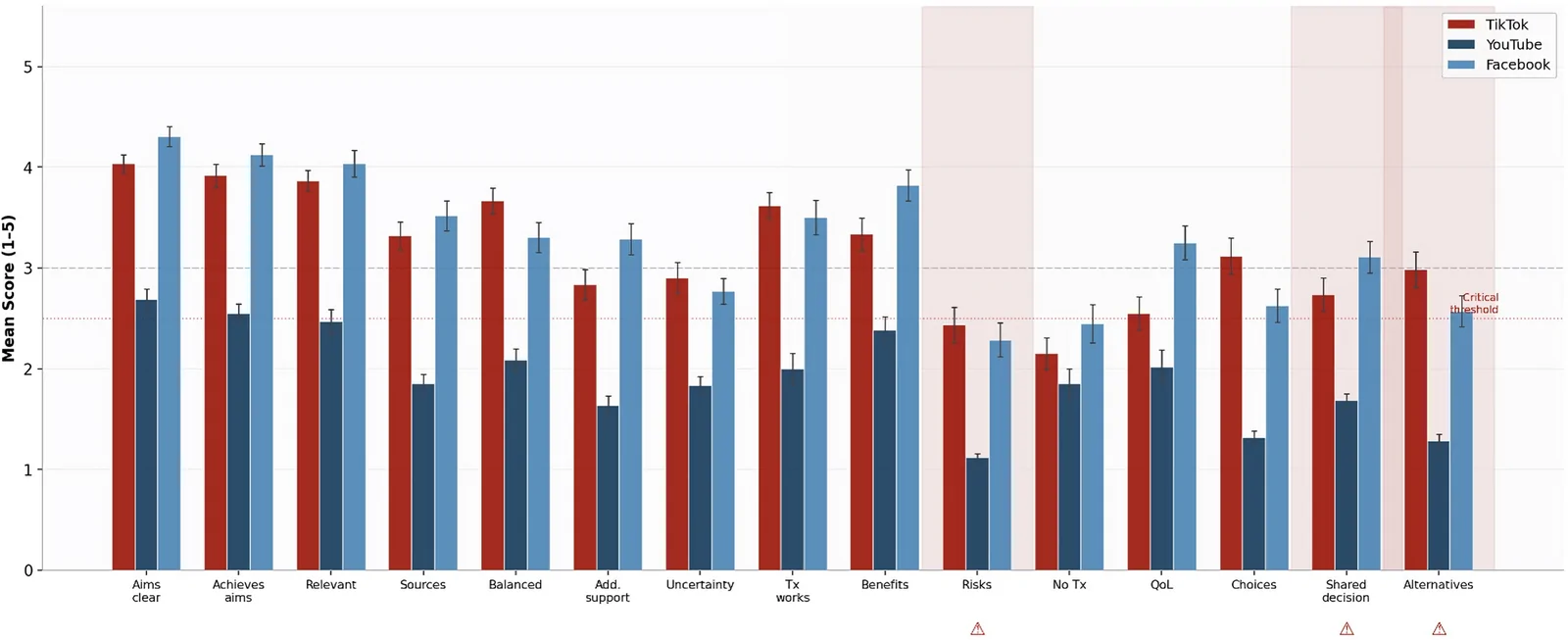
DISCERN Item-Level Scores by Platform (Bar Chart)

**Fig. 3:**
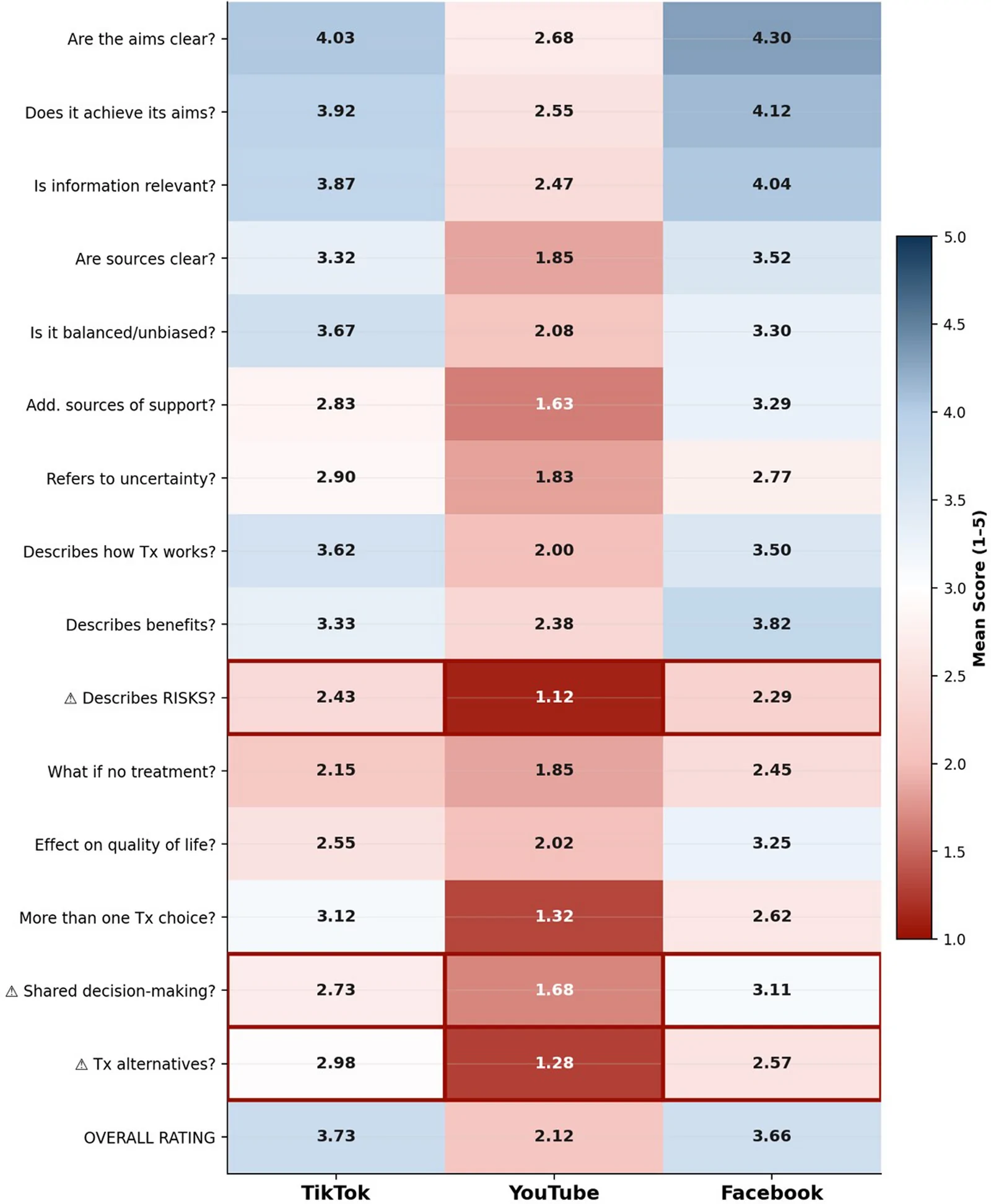
DISCERN Item Completeness Heatmap

Systematic gaps were identified across all platforms. The most critically deficient items were risk disclosure (overall mean 1.94/5), consequences of no treatment (2.14/5), treatment alternatives (2.27/5), and support for shared decision-making (2.49/5). YouTube scored lowest on every item. Facebook achieved the highest scores for clarity of aims (4.30/5) and achievement of aims (4.12/5; Table 5; Figs. 2 and 3).

**Table 5:** Mean DISCERN Item Scores by Platform (1–5; Averaged Across 4 Raters)

#	DISCERN Item	TikTok	YouTube	Facebook	Overall
1	Are the aims clear?	4.03	2.68	4.30	3.66
2	Does it achieve its aims?	3.92	2.55	4.12	3.52
3	Is information relevant to patients?	3.87	2.47	4.04	3.44
4	Are sources of information clear?	3.32	1.85	3.52	2.88
5	Is information balanced and unbiased?	3.67	2.08	3.30	3.01
6	Details of additional sources of support?	2.83	1.63	3.29	2.57
7	Does it refer to areas of uncertainty?	2.90	1.83	2.77	**2.49**
8	Does it describe how treatment works?	3.62	2.00	3.50	3.03
9	Does it describe benefits of treatment?	3.33	2.38	3.82	3.16
10	⚠** Does it describe RISKS of treatment?**	2.43	1.12	2.29	**1.94**
11	What happens if no treatment is used?	2.15	1.85	2.45	**2.14**
12	Does it describe effect on quality of life?	2.55	2.02	3.25	2.59
13	Is it clear there is more than one Tx choice?	3.12	1.32	2.62	**2.35**
14	⚠** Does it support SHARED DECISION-MAKING?**	2.73	1.68	3.11	**2.49**
15	⚠** Does it provide information on Tx ALTERNATIVES?**	2.98	1.28	2.57	**2.27**
—	DISCERN Overall Rating	3.73	2.12	3.66	3.16

⚠ = critical patient-safety items with overall mean < 2.5/5. Highlighted rows (pale red) = DISCERN domains essential to informed consent and shared decision-making. Scores 1 (not addressed) to 5 (fully addressed)

### Engagement Metrics Across Platforms

Total views differed significantly (*H* = 33.84, *p* < 0.001): TikTok median 3,000,000 > YouTube median 859,287 (*p*Bonf = 0.002, *r* = − 0.73) > Facebook median 48,000 (all pairwise *p*Bonf < 0.001). Total engagement was even more divergent (*H* = 38.45, *p* < 0.001): TikTok 305,300 vs. YouTube 8,965 vs. Facebook 147 (all pairwise *r* ≥ 0.96, *p*Bonf < 0.001). Engagement rate (*H* = 29.04, *p* < 0.001): TikTok 10.3% vs. YouTube 0.64% vs. Facebook 0.30%; TikTok exceeded both (*p*Bonf < 0.001); YouTube and Facebook did not differ (*p*Bonf = 0.54).

### Professional vs. Non-Professional Uploaders

**Fig. 4:**
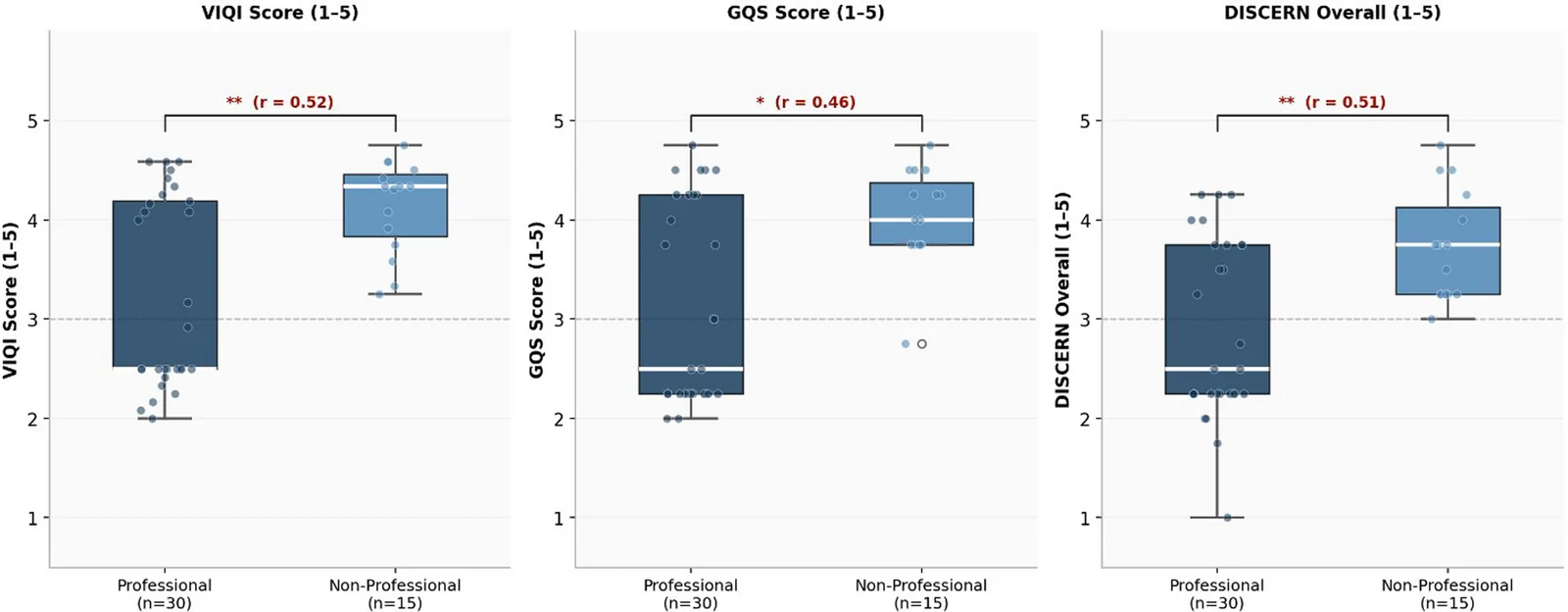
Quality Scores by Uploader Professional Status

Non-professional uploaders (*n* = 15; all TikTok) achieved significantly higher VIQI scores (median 4.33 vs. 2.50; *U* = 104, *p* = 0.005, *r* = 0.52), GQS scores (median 4.00 vs. 2.50; *U* = 118, *p* = 0.013, *r* = 0.46), and DISCERN overall ratings (median 3.75 vs. 2.50; *U* = 106, *p* = 0.006, *r* = 0.51) compared with professional uploaders (*n* = 30) (Fig. 4). Conversely, professionals achieved dramatically lower engagement: total views (median 172,492 vs. 3,000,000; *r* = 0.87), total engagement (912 vs. 305,300; *r* = 1.00), and engagement rate (0.6% vs. 10.3%; *r* = 1.00) (Table 6).

**Table 6:** Professional vs. Non-Professional Uploaders: Quality and Engagement Comparison

Variable	Professional Median (*n* = 30)	Non-Professional Median (*n* = 15)	U	*p*	*r* (effect size)
VIQI Score (1–5)	2.50	4.33	104	0.005	0.52
GQS Score (1–5)	2.50	4.00	118	0.013	0.46
DISCERN Overall (1–5)	2.50	3.75	106	0.006	0.51
Total Views	172,492	3,000,000	30	< 0.001	0.87
Total Engagement	912	305,300	0	< 0.001	1.00
Engagement Rate	0.60%	10.3%	0	< 0.001	1.00

r = effect size = 1 − 2U/(n1 × n2). Professional = hospital, healthcare institution, official medical media, or physician. All TikTok uploaders are non-professional; all YouTube and Facebook are professional — platform confounding should be considered

### Correlations: Quality, Engagement, and Video Characteristics

**Fig. 5:**
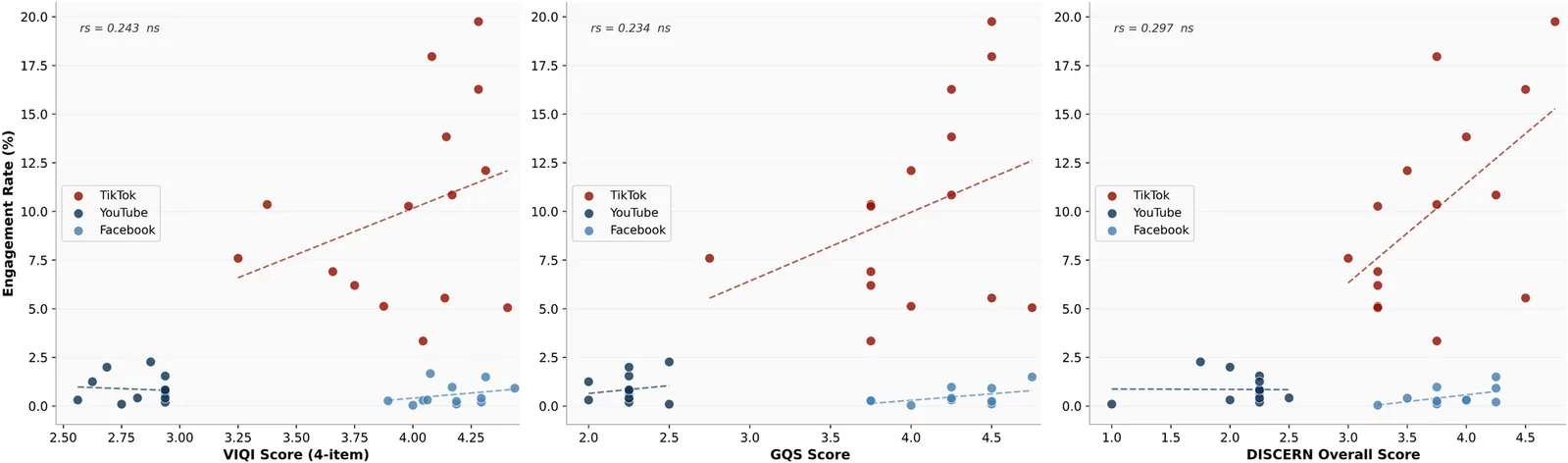
Information Quality vs. Engagement Rate by Platform

VIQI, GQS, and DISCERN overall were mutually strongly correlated (VIQI vs. GQS: *r*s = 0.934; VIQI vs. DISCERN: *r*s = 0.884; GQS vs. DISCERN: *r*s = 0.855; all *p* < 0.001) (Fig. 5). No quality metric significantly predicted total views or total engagement (all *p* > 0.43). VIQI marginally predicted engagement rate (*r*s = 0.309, *p* = 0.049). All quality scales were inversely associated with video duration, follower count, and video age, largely reflecting platform-level confounding (Table 7).

**Table 7:** Spearman Rank Correlations Between Quality Scales, Engagement, and Video Characteristics (*N* = 45)

Variable Pair	VIQI rs	*p*	GQS rs	*p*	DISCERN rs	*p*
VIQI vs. GQS	0.934	< 0.001	—	—	—	—
vs. DISCERN Overall	0.884	< 0.001	0.855	< 0.001	—	—
vs. Total Views	-0.087	0.575	-0.121	0.434	-0.094	0.543
vs. Total Engagement	0.013	0.931	—	—	0.038	0.807
vs. Engagement Rate	0.309	0.049	0.234	0.141	0.297	0.059
vs. Video Duration	-0.470	0.001	-0.442	0.003	-0.439	0.003
vs. Follower Count	-0.539	< 0.001	-0.533	< 0.001	-0.521	< 0.001
vs. Video Age (days)	-0.589	< 0.001	-0.648	< 0.001	-0.611	< 0.001

*rs * Spearman rank correlation. Red = significant at α = 0.05 (two-tailed). “—” = not computed. Negative associations with duration/age/followers largely reflect platform-level confounding

### Qualitative Analysis of Rater Notes

#### Approach

A thematic analysis was conducted on all substantive rater comments extracted from rater notes and free-text fields across four raters. Comments rated as trivially empty were excluded, yielding 47 substantive comments. Inductive analysis identified five overarching themes (Table 8).

**Table 8:** Qualitative Themes from Rater Comments: Summary and Representative Excerpts

#	Theme	Platform(s)	Description	Prevalence	Representative Rater Excerpts
1	Visual storytelling without clinical depth	TikTok	Silent visual-only format; no spoken clinical explanations; animations may be inaccurate	15/15 TikTok	*“no audio*,* just diagrammatic appearance of RYGB”; “The animations provide totally wrong expectations"*
2	Surgical transformation as sole message	YouTube & TikTok	Videos reduced to weight/body-profile changes; no clinical context	All 15 YouTube; most TikTok	*“Only changes in weight and profile reported” (Rater 4); “motivation — no any informations” (Rater 1)*
3	Incomplete risk & outcome disclosure	All platforms	Absence of complications, long-term outcomes, and post-operative risks	10 + videos across platforms	*“No complications provided”; “Deficit in long-term complications”; “only 4 months follow-up"*
4	Promotional and advertorial framing	Facebook	Physician self-promotion, surgical tourism marketing, lack of balanced clinical perspective	4–5 Facebook videos	*“advertising for surgical tourism”; “just doctor advertisement”; “advertising way"*
5	Accessibility and emotional appropriateness	TikTok & Facebook	Non-English content; patient distress footage; graphic surgical content without warnings	2–3 videos	*“not in English — could not evaluate”; “fear of the patient — not suitable for all patients"*

#### Themes

- **Theme 1: Visual Storytelling Without Clinical Depth (TikTok)**
  - All 15 TikTok videos relied exclusively on visual content — photographs, transformation timelines, and surgical animations — without spoken clinical commentary. Raters noted: *“no audio*,* just diagrammatic appearance of RYGB”*; *“very good for patient motivation*,* but no clinical information”*; *“The animations at some points provide totally wrong expectations.”* A recurring structural observation: *“Should be divided into smaller clips”* (noted independently for four separate videos).
- **Theme 2: Surgical Transformation as Sole Message (YouTube / TikTok)**
  - Rater 4 provided near-identical commentary for all 15 YouTube videos: *“Only changes in weight and profile were reported.”* Rater 1 similarly described TikTok content as *“motivational to patients without information”* or *“pure motivation — shape changes only*,* no any informations.”* This convergence across independent raters confirms that YouTube videos in the sample functioned as visual testimonials rather than clinical education, despite institutional origins.
- **Theme 3: Incomplete Risk and Outcome Disclosure (All Platforms)**
  - Multiple raters across platforms flagged absent complications, risks, and long-term outcomes: *“No complications provided”* (TikTok); *“only preoperative and after 4 months — no long-term follow-up”* (YouTube); *“Deficit in long-term complications”* (TikTok, two separate videos). The sole negative-tone Facebook documentary was noted as *“perfect in describing the journey of complications or leak”*, implicitly marking it as the exception rather than the norm.
- **Theme 4: Promotional and Advertorial Framing (Facebook)**
  - Several Facebook videos were identified as promotional: *“advertising for surgical tourism — journey*,* enjoying”*; *“just doctor advertisement — very simple and short video”*; *“Advertising way.”* At least one Facebook video featured a “carpenter/tool analogy” to explain bariatric surgery — described by a rater as creative but potentially misleading.
- **Theme 5: Accessibility and Emotional Appropriateness (TikTok / Facebook)**
  - Two sub-themes emerged. First, language barriers: “not in English so could not evaluate.” Second, emotional appropriateness: “the fear of the patient was a significant issue — not suitable for all patients.” Additionally: “pure surgery and its details — not for all patients to see” (noted on two TikTok videos featuring graphic surgical footage without discretion warnings).

## Discussion

Video content on MBS has rapidly expanded over the past decade, paralleling the increasing global uptake of these procedures. This growth has been further amplified by the use of such content as a form of advertisement and patient engagement [14, 15]. Consequently, there is a pressing need for systematic evaluation of this material to ensure its reliability, as it plays a significant role in shaping patient decisions and attitudes toward surgery. Our comprehensive study provides a cross-platform evaluation of MBS-related video content on TikTok, YouTube, and Facebook, integrating quantitative quality assessment with qualitative thematic analysis to better understand how surgical information is presented and consumed within contemporary digital environments.

A total of 45 videos were analyzed, evenly distributed across TikTok, YouTube, and Facebook. Video duration differed significantly across platforms (H = 26.18, *p* < 0.001), with TikTok videos being markedly shorter (mean 13.9 ± 6.0 s) compared to Facebook (168.9 ± 239.7 s) and YouTube (899.0 ± 1485.9 s). Despite their brevity, TikTok videos achieved the highest engagement, with a median of 3,000,000 views and an engagement rate of 10.3%, significantly exceeding YouTube (859,287 views; 0.64%) and Facebook (48,000 views; 0.30%) (all *p* < 0.001). Content characteristics also differed substantially: all TikTok videos were non-professional, patient-experience–based, and positively framed, whereas all YouTube and Facebook videos were produced by professional healthcare sources, with predominantly educational intent.

Information quality demonstrated a consistent platform-dependent pattern. The VIQI composite score differed significantly (*H* = 29.29, *p* < 0.001), with TikTok (4.14 ± 0.47) and Facebook (4.13 ± 0.51) outperforming YouTube (2.33 ± 0.18). A similar trend was observed across GQS (*H* = 29.99, *p* < 0.001) and DISCERN overall scores (*H* = 29.44, *p* < 0.001), where TikTok and Facebook achieved comparable ratings (GQS ≈ 4.0; DISCERN ≈ 3.7), while YouTube consistently ranked lowest (GQS 2.25; DISCERN 2.12). High-quality content (GQS ≥ 4) was present in 60.0% of TikTok and 66.7% of Facebook videos, but absent in YouTube (0%). Strong correlations were observed between all quality metrics (rs ≥ 0.855, *p* < 0.001), confirming internal consistency.

However, critical informational gaps were identified across all platforms. DISCERN item-level analysis revealed poor coverage of essential patient-centered domains, including risk disclosure (mean 1.94/5), consequences of no treatment (2.14/5), and treatment alternatives (2.27/5). Notably, no quality metric significantly predicted total views or engagement (all *p* > 0.43), indicating that content visibility operates independently of informational quality. Furthermore, non-professional content (all TikTok) demonstrated significantly higher quality scores than professional content (*p* ≤ 0.013), while simultaneously achieving dramatically greater reach and engagement. These findings highlight a pronounced mismatch between content dissemination, perceived value, and the delivery of comprehensive, clinically relevant information.

This mixed-methods cross-platform analysis provides a comprehensive evaluation of MBS related video content on TikTok, YouTube, and Facebook, revealing several critical insights regarding information quality, reliability, and audience engagement. The findings demonstrate a striking divergence between informational quality and user engagement, highlight substantial deficiencies in clinically essential content domains, and underscore the influence of platform-specific content structures on how bariatric surgery information is communicated and perceived. 

A principal finding of this study was the lack of a clear association between audience reach and informational quality. TikTok videos achieved substantially higher numbers of views, total engagement, and engagement rates than YouTube and Facebook videos; however, total views and total engagement were not significantly associated with VIQI, GQS, or DISCERN scores. VIQI showed only a weak positive association with engagement rate (rs = 0.309, p = 0.049). This aligns with prior literature demonstrating that social media engagement is driven primarily by entertainment value, emotional resonance, and visual appeal rather than informational accuracy or completeness [16]. The absence of correlation between quality metrics and engagement reinforces concerns that widely disseminated content may not reflect high-quality or evidence-based information. In the context of MBS, this disconnect is particularly important, as patient expectations and decision-making may be shaped by highly visible yet incomplete or misleading content.

Interestingly, TikTok videos demonstrated high VIQI and GQS scores despite the absence of spoken explanations and the exclusive reliance on visual storytelling. This finding appears counterintuitive when compared with traditional assumptions that longer, professionally produced educational content provides superior informational value. However, the VIQI instrument partially captures flow, visual clarity, and presentation quality, which may favor concise and visually structured content typical of TikTok [17]. Nevertheless, qualitative analysis revealed that these videos frequently lacked clinical depth, omitted essential information, and occasionally conveyed unrealistic expectations regarding surgical outcomes. This discrepancy highlights a limitation of quantitative scoring systems when applied to short-form media and emphasizes the importance of complementary qualitative assessment.

The dominance of patient-experience narratives on TikTok and, to a lesser extent, YouTube reflects a broader trend in social media health communication, where personal storytelling is often prioritized over evidence-based education. While such narratives may enhance relatability and patient engagement, they inherently lack generalizability and may introduce bias through selective outcome presentation. Previous studies have shown that patient testimonials often emphasize positive outcomes while underreporting complications and long-term challenges, potentially leading to unrealistic expectations among viewers [18]. In the present study, this phenomenon was consistently observed, with transformation-focused content serving as the primary message across many videos.

The underrepresentation of shared decision-making elements further highlights a gap between available online content and contemporary patient-centered care principles. High-quality health information should support patients in understanding available options, weighing risks and benefits, and participating actively in treatment decisions. However, the majority of videos analyzed in this study did not adequately address these aspects, suggesting that current social media content may not effectively support informed decision-making processes. This finding is particularly relevant given evidence that educational interventions, including video-based tools, can significantly enhance patient understanding and engagement during the informed consent process [19–21].

Platform-specific differences in content characteristics also provide important insights. YouTube and Facebook videos were predominantly produced by healthcare professionals and institutions; yet, paradoxically, they demonstrated lower quality scores than TikTok videos. This result may be explained by the nature of the included content, which often consisted of transformation-focused or promotional material rather than structured educational presentations. Prior research has similarly noted that the presence of a healthcare professional does not guarantee high-quality or comprehensive information, particularly in unregulated online environments [22].

Facebook content, while less engaging overall, demonstrated a higher prevalence of healthcare professionals on screen and spoken explanations, suggesting a more traditional educational format. However, qualitative analysis identified a subset of videos with clear promotional intent, including surgical tourism advertising and physician self-promotion. The presence of such content raises concerns regarding conflicts of interest and the potential for biased information dissemination. These findings are consistent with broader concerns about commercialization in online health information, where promotional objectives may overshadow educational value [23].

The inverse relationship observed between video duration and quality scores further reflects the influence of platform-specific characteristics. Shorter videos, particularly on TikTok, were associated with higher quality scores, whereas longer YouTube videos demonstrated lower scores. This may be attributed to differences in content structure, viewer retention strategies, and algorithmic preferences. Short-form content often necessitates concise and visually optimized messaging, which may enhance perceived clarity and flow [24]. However, this brevity inherently limits the ability to convey complex medical information comprehensively, reinforcing the need for cautious interpretation of quality metrics in this context.

The qualitative findings provide critical context to the quantitative results and reveal recurring patterns that are not fully captured by standardized scoring systems. The theme of “visual storytelling without clinical depth” underscores the limitations of visually driven content in conveying nuanced medical information. Similarly, the identification of “incomplete risk and outcome disclosure” across platforms highlights a systemic issue in online bariatric surgery content. These findings emphasize that while quantitative tools such as VIQI, GQS, and DISCERN are valuable, they should be complemented by qualitative analyses to achieve a more comprehensive evaluation.

A further notable observation is the emotional framing of content, particularly on TikTok and Facebook. Videos highlighting dramatic transformations, patient fear, or surgical experiences may shape viewer perceptions through emotional engagement rather than objective clinical information. This aligns with existing literature suggesting that emotionally charged content is more likely to be shared and engaged with on social media platforms [25]. While such content may enhance awareness and outreach, it may also distort public understanding of the risks and benefits associated with bariatric surgery [26, 27]. In addition, Rukavina et al. reported frequent unprofessional behavior among healthcare professionals on social media, including breaches of patient privacy and blurred professional boundaries, alongside insufficiently robust regulatory guidelines governing online conduct [28]. Collectively, these factors may contribute to a distorted representation of bariatric surgery within social media environments.

The findings of this study have important clinical implications. Given the widespread use of social media as a source of health information, healthcare professionals must recognize its influence on patient knowledge and expectations. The observed deficiencies in content quality and completeness highlight the need for greater involvement of healthcare professionals in producing accurate, balanced, and accessible educational content. Additionally, there is a need for strategies to improve the visibility of high-quality information, potentially through platform-level interventions or collaboration with content creators.

Importantly, the health-information landscape is rapidly evolving, and patients are increasingly turning not only to social-media platforms but also to large language models (LLMs) and other artificial intelligence–based tools to obtain information about diseases, treatment options, and surgical procedures [29]. This emerging pattern introduces a new dimension to digital health communication, as patients may use LLM-generated responses alongside or instead of conventional online sources when seeking medical advice. Although LLMs may provide accessible and personalized explanations, their responses can also reflect limitations in the underlying training data and may contain inaccurate, incomplete, or insufficiently contextualized information. Therefore, the deficiencies identified in social-media content should be considered within this broader transition toward AI-mediated health information seeking. The observed deficiencies in content quality and completeness highlight the need for greater involvement of healthcare professionals in producing accurate, balanced, and accessible educational content [30]. Additionally, there is a need for strategies to improve the visibility of high-quality information, potentially through platform-level interventions, collaboration with content creators, and the development of reliable AI-assisted patient education resources [31].

From a research perspective, this study contributes to the growing body of literature evaluating online health information by providing a comparative analysis across multiple platforms and integrating both quantitative and qualitative methodologies. The use of validated assessment tools alongside thematic analysis enhances the robustness of the findings and allows for a more nuanced understanding of content characteristics.

Several limitations should be acknowledged. The sample size was limited to 45 videos, and only the top-ranked results were included, which may not fully represent the diversity of available content. Although we selected TikTok, YouTube, and Facebook because they are among the most widely used and influential social-media platforms for health-related content, other platforms were not evaluated, and their content may demonstrate different patterns of quality and engagement. Furthermore, some videos appeared to be produced by individuals who were not readily identifiable as bariatric surgeons or healthcare professionals. However, to minimize selection bias and reflect the content that users may realistically encounter on these platforms, videos were selected using a predefined random sampling approach rather than selectively excluding content based on the uploader’s professional background. Additionally, the perfect confounding between platform and uploader type limits the ability to isolate the independent effects of professional status. Finally, although validated assessment tools were used, their applicability to short-form video content may be limited, as previously discussed.

Future research should explore larger and more diverse samples, including additional platforms, and examine the impact of social-media content on patient knowledge, attitudes, and clinical outcomes. The integration of artificial intelligence–based content analysis may also provide new opportunities for large-scale evaluation of online health information.

## Conclusion

This study demonstrates that MBS-related content on social media platforms is characterized by high engagement but variable and often insufficient informational quality. The lack of correlation between engagement and quality, combined with significant deficiencies in critical educational domains, underscores the need for improved content standards and greater involvement of healthcare professionals in digital health communication. Addressing these challenges is essential to ensure that social media serves as a reliable and effective tool for patient education in bariatric surgery.

## Supplementary Information

Below is the link to the electronic supplementary material.

Supplementary Material 1 (DOCX 19.1 KB)

## Data Availability

No datasets were generated or analysed during the current study.

## Declarations

### Artificial Intelligence Use

No artificial intelligence tools were used in the preparation of this manuscript.
