# Supplementary material for: Quality, Reliability, and Content Characteristics of Metabolic Bariatric Surgery Videos on TikTok, YouTube, and Facebook: A Mixed-Methods Cross-Platform Analysis
Source: Obes Surg. 2026 Sep 11;36(10):5404–19. doi: 10.1007/s11695-026-08922-9 (PMC13645848; doi:10.1007/s11695-026-08922-9)
Supplement: Supplementary file 1 [file 11695_2026_8922_MOESM1_ESM.docx]

**Supplementary materials**

**Table S1: The analyzed videos retrieved from TikTok, Facebook, and YouTube**

| **Platform** | **Video Title** | **Video URL** |
| --- | --- | --- |
| TikTok | Bariatric surgery | <https://vt.tiktok.com/ZS5wGb7rC/> |
| TikTok | Bariatric surgery | <https://vt.tiktok.com/ZS5w7266M/> |
| TikTok | Gastric sleeve surgery | <https://vt.tiktok.com/ZS5wwRwHx/> |
| TikTok | Bariatric surgery | <https://vt.tiktok.com/ZS5wcL7LM/> |
| TikTok | Bariatric surgery | <https://vt.tiktok.com/ZS5wwHD6N/> |
| TikTok | Bariatric surgery | <https://vt.tiktok.com/ZSaJkBgAo/> |
| TikTok | Bariatric surgery | <https://vt.tiktok.com/ZS5E3CEHm/> |
| TikTok | Bariatric surgery | <https://vt.tiktok.com/ZS5E386UK/> |
| TikTok | 4 months gastric sleeve transformation | <https://vt.tiktok.com/ZS5wKgDey/> |
| TikTok | 5 months post VSG | <https://vt.tiktok.com/ZSaJdqWr4/> |
| TikTok | Vertical sleeve gastrectomy | <https://vt.tiktok.com/ZS5Ecrkrh/> |
| TikTok | Bariatric surgery | <https://vt.tiktok.com/ZS5w3uDBx/> |
| TikTok | Bariatric surgery | <https://vt.tiktok.com/ZSaJ81WJ5/> |
| TikTok | Before and after gastric sleeve | <https://vt.tiktok.com/ZSaJ83P2P/> |
| TikTok | Gastric sleeve | <https://vt.tiktok.com/ZSaJNaka3/> |
| You tube | Bariatric surgery | <https://www.youtube.com/watch?v=I8D8zpLqpw8> |
| You tube | Gastric Bypass Surgery \| Duke Health | <https://www.youtube.com/watch?v=ErDTNYtDcAQ> |
| You tube | Roux-en-Y Gastric bypass | <https://www.youtube.com/watch?v=z8Ajg53fpkA> |
| You tube | Sleeve Gastrectomy Demonstration: Memorial Weight-Loss Surgery Program | <https://www.youtube.com/watch?v=ExC83UdR0X0&rco=1> |
| You tube | Laparoscopic Sleeve Gastrectomy Video - Brigham and Women's Hospital | <https://www.youtube.com/watch?v=KM6UQzMwbWU&rco=1> |
| You tube | Gastric Bypass Surgery Demonstration: Memorial Weight-Loss Surgery Program | <https://www.youtube.com/watch?v=gwVnGs9p-h0&rco=1> |
| You tube | Bariatric Surgery \| Types of surgery \| 2/3 | <https://www.youtube.com/watch?v=Z7xExtOtI9w> |
| You tube | Bariatric Surgery Full Liquid Diet | <https://www.youtube.com/watch?v=LTYy4NWVz1M> |
| You tube | Gastric Bypass Procedure Animation \| J&J MedTech | <https://www.youtube.com/watch?v=x_w4CdEl67s> |
| You tube | Is Ozempic Putting An End To Bariatric Surgery? \| Dr. Christine Ren-Fielding | <https://www.youtube.com/watch?v=xycL2kqg5is> |
| You tube | What Happens During the Endoscopic Sleeve Gastroplasty (ESG) Procedure? | <https://www.youtube.com/watch?v=juqqo7PQWEs> |
| You tube | At Over 660-lbs, Ashley Learns the Risks of Bariatric Surgery \| My 600-lb Life | <https://www.youtube.com/watch?v=H4yoN9oq8wY> |
| You tube | Weight Loss Surgery: Robotic Bariatric Procedure | <https://www.youtube.com/watch?v=55S-JleEoeU> |
| You tube | Eating After Bariatric Surgery - A guide for the first month | <https://www.youtube.com/watch?v=J420pX3vfpI> |
| You tube | Gastric Bypass vs Sleeve, update for 2021 | <https://www.youtube.com/watch?v=F5YDrqon7G0> |
| Facebook | Is Bariatric Surgery a Weight-Loss Shortcut? | https://www.facebook.com/share/r/1AM1AeybUw/ |
| Facebook | How Bariatric Surgery Lowered Heart Risk and Increased Activity | https://www.facebook.com/share/v/18NDciS5nd/ |
| Facebook | Bariatric Surgery Testimony | https://www.facebook.com/share/v/1AtLCkEZtu/ |
| Facebook | Bariatric Surgery with Dr. Michael Murr | https://www.facebook.com/share/v/184QohGf5J/ |
| Facebook | Dr. Latzko's Top 5 Benefits of Bariatric Surgery | https://www.facebook.com/share/v/188ew8ZTth/ |
| Facebook | Dr. Thatimatla on the Benefits of Bariatric Surgery | https://www.facebook.com/share/v/1814v2t4Kh/ |
| Facebook | Bariatric Surgery | https://www.facebook.com/share/v/1DJphWgyNQ/ |
| Facebook | Top 5 Benefits of Bariatric Surgery | https://www.facebook.com/share/v/15r7S8s473/ |
| Facebook | My Bariatric Surgery in Mexico | https://www.facebook.com/share/v/1Tju4Qb56T/ |
| Facebook | Robotic Bariatric Surgery II Weight loss surgery | https://www.facebook.com/share/v/1AQ7fphc4e/ |
| Facebook | What Are the Benefits of Bariatric Surgery? | https://www.facebook.com/share/v/1BqbtXEmb9/ |
| Facebook | The bariatric surgery which turned into a nightmare battle to stay alive | https://www.facebook.com/share/v/1QPWV9CNAK/ |
| Facebook | 💬 Thinking about Bariatric Surgery? | https://www.facebook.com/share/v/1FAuXpb7JM/ |
| Facebook | She Got Bariatric Surgery at 15 Years Old | https://www.facebook.com/share/v/1C9gVxwDBX/ |
| Facebook | When to Consider Bariatric Surgery with Dr. Venkat | https://www.facebook.com/share/v/1DtYV75oRh/ |
